# Mycobiota in the Carposphere of Sour and Sweet Cherries and Antagonistic Features of Potential Biocontrol Yeasts

**DOI:** 10.3390/microorganisms9071423

**Published:** 2021-06-30

**Authors:** Ramunė Stanevičienė, Juliana Lukša, Živilė Strazdaitė-Žielienė, Bazilė Ravoitytė, Regina Losinska-Sičiūnienė, Raimondas Mozūraitis, Elena Servienė

**Affiliations:** 1 Laboratory of Genetics, Institute of Botany, Nature Research Centre, Akademijos str. 2, LT-08412 Vilnius, Lithuania; ramune.staneviciene@gamtc.lt (R.S.); juliana.luksa@gamtc.lt (J.L.); zivile.strazdaite-zieliene@gamtc.lt (Ž.S.-Ž.); bazile.ravoityte@gamtc.lt (B.R.); regina.losinska-siciuniene@gamtc.lt (R.L.-S.); 2 Laboratory of Chemical and Behavioral Ecology, Institute of Ecology, Nature Research Centre, Akademijos str. 2, LT-08412 Vilnius, Lithuania; raimondas.mozuraitis@gamtc.lt

**Keywords:** *P. cerasus*, *P. avium*, cherry, yeasts, fungal communities, antagonistic activity

## Abstract

Sour cherries (*Prunus cerasus* L.) and sweet cherries (*P. avium* L.) are economically important fruits with high potential in the food industry and medicine. In this study, we analyzed fungal communities associated with the carposphere of sour and sweet cherries that were freshly harvested from private plantations and purchased in a food store. Following DNA isolation, a DNA fragment of the ITS2 rRNA gene region of each sample was individually amplified and subjected to high-throughput NGS sequencing. Analysis of 168,933 high-quality reads showed the presence of 690 fungal taxa. Investigation of microbial ASVs diversity revealed plant-dependent and postharvest handling-affected fungal assemblages. Among the microorganisms inhabiting tested berries, potentially beneficial or pathogenic fungi were documented. Numerous cultivable yeasts were isolated from the surface of tested berries and characterized by their antagonistic activity. Some of the isolates, identified as *Aureobasidium pullulans*, *Metschnikowia fructicola*, and *M. pulcherrima*, displayed pronounced activity against potential fungal pathogens and showed attractiveness for disease control.

## 1. Introduction

Sour cherries (*Prunus cerasus* L.) and sweet cherries (*P. avium* L.) belong to the family of the *Rosaceae* [1] and are economically and agronomically important crops. These species are cultivated in temperate and cool regions and characterized by a scattered distribution from the Black Sea to Ireland and Spain, and from Scandinavia to Africa [2]. Cherry fruits are a nutrient-dense food with relatively low caloric content, important nutrients, and significant amounts of bioactive food components, having positive effects on human health [3].

Sour cherry is cultivated for its sharp and juicy fruits that are mostly destined to produce foods like jam, jelly, and syrup or alcohol beverages such as wine, brandy, and fruit beer [1,4]. The current research suggests that intake of fresh sour cherry berries or juice promotes health due to the positive action of anthocyanins and phenolic compounds [3,5]. The consumption of cherries may reduce the risk of arthritis, cancer, cardiovascular disease, and diabetes, and may improve sleep and cognitive functions [3].

Sweet cherry is one of the most economically important fruit species in the world. Most sweet cherry fruits are consumed fresh or processed as frozen, dried, or juiced [3]. Sweet cherry berries are rich in bioactive compounds, including flavonoids, anthocyanins, carotenoids, vitamins, ascorbic acid, and potassium [6,7]. Both laboratory assays and clinical trials have demonstrated the anti-inflammatory, antimicrobial, and anticarcinogenic properties of sweet cherry fruits and their metabolites [6,8].

The plant carposphere is highly colonized by various bacterial and fungal microorganisms, the distribution of which is affected by geographic location, climatic conditions, plant species, ripening stage, and growing methods [9,10,11]. Some epiphytic plant-associated microorganisms demonstrate beneficial features, produce secondary metabolites improving resistance in the plant, and impact the structure of the microbial population [12,13,14]. On the other hand, some fruit-inhabiting microorganisms are recognized as pathogenic to hosting plants and humans and are responsible for significant economic losses and serious health problems [15,16]. Therefore, postharvest handling and processing of fruits encounter exceptional attention due to the control of microbiological hazards [17,18].

The scarcity of works characterizing sweet and sour cherries microbial communities are of certain importance. Based on high-throughput sequencing of the 16S rRNA gene, the nitrogen-fixing bacterial community inhabiting *Prunus avium* L. leaves was described [19] and bacterial diversity of pitted sweet cherries, proceeded high-hydrostatic pressure processing, was evaluated [20]. Endophytic fungi associated with *Prunus avium* and *Prunus cerasus* trees located in Germany and Iran were isolated and examined by morphological and molecular analysis [21,22]. Microorganisms present on different cultivars of sour cherries in Hungary were evaluated using cultivation techniques only [18]. It was demonstrated that the frequency of distribution of bacteria, molds, and yeasts were very similar in spite of different cultivars, growing methods, and growing years [18]. To the best of our knowledge, there is no information on sweet and sour cherries carposphere-associated fungal microorganism communities.

The main objectives of this study were: (i) to characterize the fungal communities distributed on freshly harvested *P. avium* and *P. cerasus* berries and compare with a food store sour cherry, (ii) to isolate and identify the cultivable yeasts associated with sweet and sour cherries to explore their biocontrol potential, and (iii) to assess the antagonistic activity of isolated fungi against other yeasts, including potential pathogens. We aim to better understand the abundance of fungi on the surface of cherries and evaluate the differences between the fungal communities. This research employs natural resources to explore the properties of environmental fungi and dedicates particular attention to the fight against potential pathogens. Obtained results may aid in developing eco-friendly biocontrol tools.

## 2. Materials and Methods

### 2.1. Sampling of Berries

Sweet cherries (*P. avium* L.) were harvested three times in June 2020 from 8–10-year-old sweet cherry trees located in a private plantation in the Alytus region of Lithuania (GPS coordinates: 54°23′43.8″ N, 23°56′18.7″ E). Sour cherries (*P. cerasus* L.) were sampled three times on July 2020 from the 8–12-year-old trees located in a private plantation in the Vilnius region of Lithuania (GPS coordinates: 54°45′08.2″ N, 25°17′10.0″ E). Sterile gloves and plastic bags were used to aseptically collect berries, and samples were processed in the laboratory within 3 h after harvesting. At each sampling, the berries were collected from different cherry trees and combined into one biological replicate at each location. In total, three sweet cherries samples (PA-PL1, PA-PL2, and PA-PL3) and three samples of sour cherries (PC-PL1, PC-PL-2, and PC-PL3) were collected from private plantations and prepared for further analysis. In addition, sour cherries, which originated from conventional orchards of Hungary, were purchased at the “ready-to-eat” ripening stage in June 2020 from a local food store (Vilnius, Lithuania) (samples PC-FS1, PC-FS2, and PC-FS3) and kept under refrigeration for no more than 3–4 h before processing.

### 2.2. Yeast Isolation and Culturing

To determine the viable population of yeasts distributed on sweet and sour cherries, about 25 g of the berries were placed in a 30 mL of liquid MD medium (2% dextrose, 1% (NH_4_)_2_SO_4_, 0.09% KH_2_PO_4_, 0.05% MgSO_4_, 0.023% K_2_HPO_4_, 0.01% NaCl, 0.01% CaCl_2_) for 1 h at room temperature with shaking at 100 rpm. Outwashes were serially diluted in MD medium, and 0.1 mL aliquots were plated in triplicate on yeast-extract-peptone-dextrose (YPD)-agar plates (1% yeast extract, 1% peptone, 2% dextrose, 2% agar) containing 50 μg mL^−1^ chloramphenicol for detection and enumeration of cultivable yeasts. To capture yeasts distributed in low quantities on the tested berries, the enrichment technique was used. Collected berries (15 g of each sample) were kept in 5% dextrose solution for 15 days at a temperature of 22 °C and afterward were plated on YPD-agar. Plates were incubated at 25 °C for 3–5 days and CFUs were counted. Results were expressed as log CFU/g berries. Representatives of morphologically different yeast-like colonies were purified by streaking on YPD-agar medium and subjected to molecular identification and antagonistic activity analysis.

### 2.3. Molecular Identification of Yeast Species

Genomic DNA was isolated from freshly grown yeast cells (24 h). Genomic DNA purification kit (Thermo Fisher Scientific Baltics, Vilnius, Lithuania) following the manufacturer’s instructions was employed. For the identification of yeast, PCR amplification of the region between the 18S rRNA and 28S rRNA genes containing two non-coding spacers (ITS-A and ITS-B) separated by the 5.8S rRNA gene was performed using ITS1 (5′-TCCGTAGGTGAACCTGCGG-3′) and ITS4 (5′-TCCTCCGCTTATTGATATGC-3′) primers or the D1/D2 region of 26S rDNA was amplified using NL1 (5′-GCATATCAATAAGCGGAGGAAAAG-3′) and NL4 (5′-GGTCCGTGTTTCAAGACGG-3′) primers. The PCR amplification was performed as described in [23]; except for D1/D2 region amplification, annealing temperature was 52 °C. Amplified products were purified using a GeneJet PCR purification kit (Thermo Fisher Scientific Baltics, Vilnius, Lithuania) and sequenced using ITS1 and/or NL1 primers at BaseClear (Leiden, Netherlands). The generated sequences were compared with those found in the FASTA network service of the EMBL-EBI database (http://www.ebi.ac.uk/Tools/sss/fasta/nucleotide.html (accessed on 12 May 2021) and deposited in the National Center for Biotechnology Information (NCBI) under accession numbers MZ185321-MZ185356, MZ185358-MZ185371.

### 2.4. Determination of Sour and Sweet Cherry Fungal Microbiota by Next Generation Sequencing

For metagenomic analysis, microbial suspension from the surface of cherries was obtained under aseptic conditions by washing 300 g of representative berries with 500 mL of sterile 0.05 M phosphate buffer solution (pH 6.8) for 1 h with shaking at 120 rpm. The obtained microbial cell suspension was centrifuged in 50 mL Falcon test tubes at 5000× *g* for 20 min, and the resulting pellets were collected in one Eppendorf tube, centrifuged at 12,000× *g* for 10 min, and used for DNA extraction. Genomic DNA of fungi was isolated from 40 mg of sediments obtained from respective berries using a Genomic DNA purification kit (Thermo Fisher Scientific Baltics, Vilnius, Lithuania) and following the manufacturer’s protocol. The extracted DNA was tested for quantity and purity using Nanodrop 2000 spectrophotometer (Thermo Fisher Scientific). The ITS primer pair, ITS3-KYO2 (5′-GATGAAGAACGYAGYRAA-3′) and ITS4 (5′-TCCTCCGCTTATTGATATGC-3′), was used to amplify the fungal internal transcribed spacer (ITS) DNA region ITS2. Amplicon libraries were prepared using modified Illumina adapters (www.illumina.com (accessed on 10 February 2021), and quality was checked on an Agilent Technologies Bioanalyzer DNA 1000. Amplicon sequencing was performed on the Illumina MiSeq platform to obtain paired-end reads (2 × 300 bp) (BaseClear B.V., Leiden, The Netherlands). Complete data sets were submitted to the National Center for Biotechnology Information (NCBI) Sequence Read Archive (SRA) database (Accession number PRJNA729235).

### 2.5. Bioinformatics and Data Analysis

Sequences were processed and analyzed using QIIME 2 v2020.6 open-source software https://qiime2.org (accessed on 7 July 2020) [24]. The obtained forward and reverse reads were trimmed with Cutadapt 2.8 to remove primer sequences [25]. Paired-end reads were processed with Divisive Amplicon Denoising Algorithm 2 (DADA2 pipeline http://qiime2.org (accessed on 15 February 2021). Low quality reads with an expected error rate higher than 3.7 for reverse and 2.0 for forward reads were discarded. Chimeric sequences and singletons were excluded using the ‘consensus’ method [26]. Obtained unique amplicon sequence variants (ASVs) were assigned taxonomy by aligning to the latest UNITE v8.2 dynamic reference database (build 15 January 2020) at 97% similarity using QIIME q2-feature-classifier plugin [27]. The other parameters were used as default. Fungal data for alpha and beta diversity analyses were rarefied to the minimum sequence count per berry sample (17,100) using the q2-diversity plugin. Alpha diversity was calculated by Shannon’s diversity index, observed ASVs, and Pielou’s measure of species evenness. Kruskal-Wallis test was used for taxa comparisons, calculated in QIIME 2. Permutational multivariate analysis of variance (PERMANOVA) with 999 random permutations was used to analyze statistical differences in beta diversity. Principal coordinate analysis (PCoA) plots were constructed using Jaccard distances with ggfortify tools in R v1.3.1056 [28]. Heatmap was visualized using gplot package in R [29].

### 2.6. In Vitro Evaluation of Yeast Antagonistic Activity

For detection of killing phenotype, MBA agar plates (0.5% yeast extract, 0.5% peptone, 2% dextrose, 2% agar, 0.002% methylene blue, pH 4.8) were seeded with a lawn (2 × 10^6^ cells/plate) of the sensitive *Saccharomyces cerevisiae* strain BY4741 (MATa; his3 D1; leu2D0; met15D0; ura3D0 (KIL-0)) (Thermo Scientific Molecular Biology, Lafayette, CO, USA) or strains of different yeast species (*Rhodotorula graminis*, *R. glutinis*, *Candida albicans*, *C. guilermondii*, *Sporobolomyces roseus*, *Cryptococcus wieringae*, *Aureobasidium pullulans*). The tested yeast strains were spotted on the top of the plates and followed incubation at 25 °C for 3 days. The killer activity was evaluated based on the appearance of clear zones of growth inhibition surrounding the spotted cells.

## 3. Results

### 3.1. Abundance and Diversity of Fungal Microbiota on P. cerasus and P. avium

The fungal community of sweet and sour cherry samples was revealed by Next Generation Sequencing (NGS) of the ITS2 region of fungal rDNA amplified from total DNA extracted from the surface of cherry samples. Illumina MiSeq sequencing generated a total of 257,984 raw reads. After data quality filter processing, the number of high quality ITS reads for sweet and sour cherries was 168,933 with an average of 18,770 sequences per sample (range 17,104–21,140) (Table 1). The clustering of the sequences at 97% sequence identity generated a total of 690 ASVs (Table 1). The highest number of ASVs was observed in sour cherries harvested from private plantation 317 [109 ± 5.77, hereafter median for 3 samples ± standard deviation], next followed sweet cherries 194 [71 ± 10.97], and the lowest number was in store-purchased sour cherries 179 [58 ± 14.57] (Table 1).

The mycobiota analysis according to the nonparametric Kruskal–Wallis test showed significant differences in fungal diversity amongst sour and sweet cherries collected from private plantations.

**Figure 1:**
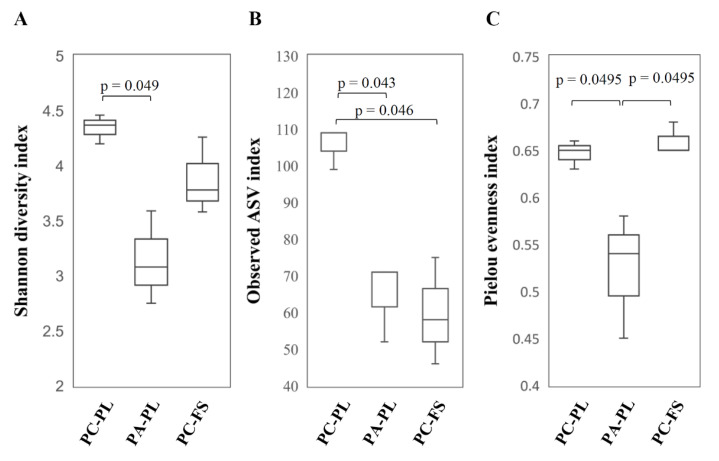
Alpha diversity analysis based on Shannon index (**A**), observed ASV index (**B**), and Pielou evenness (**C**). Samples were rarefied to sampling depth of 17,100. Kruskal-Wallis test was performed to analyze statistical significance.

Alpha diversity metrics, such as Shannon diversity index (*p* = 0.049) and Pielou’s measure of evenness index (*p*-value 0.0495) were statistically different between PC-PL and PA-PL samples (Figure 1). The observed number of ASVs revealed a significant difference in fungal community richness between PC-PL vs. PA-PL as well as PC-PL vs. PC-FS samples (ASVs index *p*-values were 0.043 and 0.046, respectively). In agreement with ASV data, Shannon’s diversity and the Simpson estimates revealed that sour cherries had higher fungal diversity than sweet cherries or food store cherries (Table 1). No statistical differences in alpha diversity metrics were detected between sweet cherries collected from the private plantation and sour cherries from a store (Figure 1).

**Figure 2:**
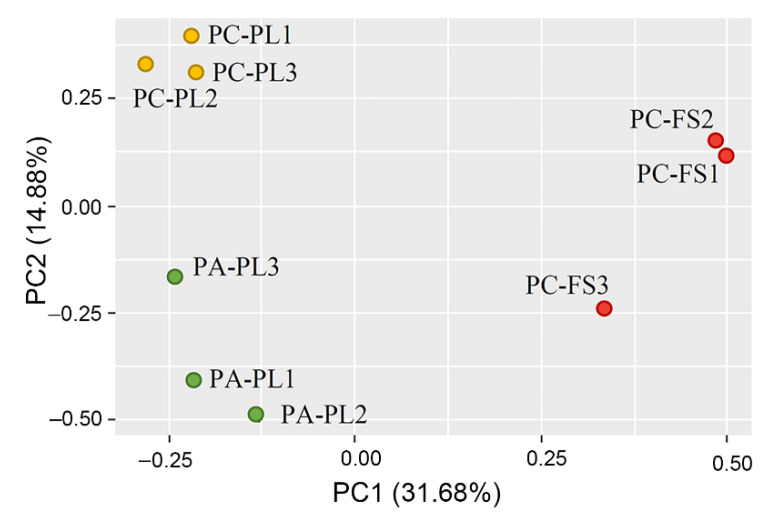
Comparison of fungal microbiota on freshly harvested sour (PC-PL) and sweet cherries (PA-PL), and sour cherries (PC-FS) from a food store by principal coordinate analysis (PCoA). Plots were calculated using Emperor unweighted UniFrac distances. Each dot represents a distinct sample.

Results from the permutational multivariate analysis of variance (pairwise PERMANOVA) showed that there are no statistically significant differences in beta diversity between PC-PL, PA-PL, and PC-FS samples (PC-PL vs. PA-PL *p* = 0.113 for Jaccard and *p* = 0.685 for Bray-Curtis; PC-PL vs. PC-FS *p* = 0.106 for Jaccard and *p* = 0.093 for Bray-Curtis; PA-PL vs. PC-FS *p* = 0.105 for Jaccard and *p* = 0.103 for Bray-Curtis). Rarefaction curves showed that all samples reached the saturation phase, and the great majority of microbial diversity was captured (Figure S1). The Principal Coordinate Analysis (PCoA) allowed clear separation of PC-PL, PA-PL, and PC-FS samples, thus pointing to differences in the fungal microbiota composition (Figure 2).

### 3.2. Fungal Community Profiling

**Figure 3:**
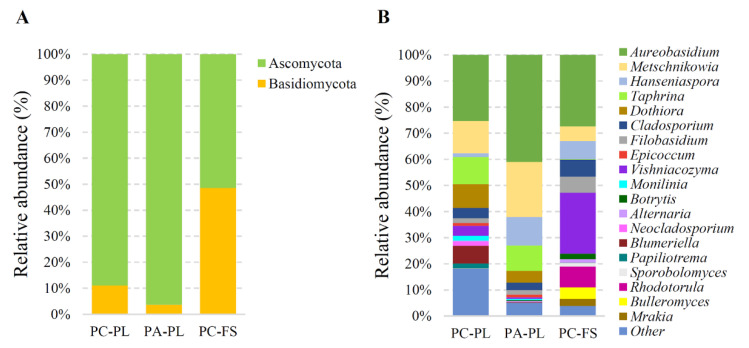
Mycobiota composition on sour (PC-PL) and sweet (PA-PL) cherries sampled from private plantations, and sour cherries (PC-FS) purchased in a food store. Relative abundance of sequences classified at the phylum (**A**) and genus (**B**) level. The taxonomic groups comprising less than 1% of the total composition were assigned to “Other”.

The metataxonomic analysis of freshly harvested sour and sweet cherries and sour cherries purchased from a local food store revealed a complex fungal microbiota. At the phylum level, the fungal microorganism composition in the freshly harvested sour and sweet cherries showed the highest relative abundance of Ascomycota (88.70% for sour cherries and 96.14% for sweet cherries), which was distributed mainly in four classes: Dothideomycetes, Taphrinomycetes, Leotiomycetes, and Saccharomycetes (Figure 3A, Table S1). The next phylum Basidiomycota (11.02% for PC-PL and 3.61% for PA-PL, respectively) was represented by fungi belonging to Tremellomycetes class. On the genus level, the most abundant genera in PC-PL and PA-PL samples were *Aureobasidium* (25.04% and 40.63%, respectively), *Metschnikowia* (12.31 and 20.96%, respectively), *Taphrina* (10.23% and 9.68%, respectively), *Dothiora* (9.04% and 4.46%, respectively), and *Cladosporium* (3.98% and 2.85%, respectively) (Figure 3B). On the freshly harvested sour cherries 66 species of fungi were identified and 42 species on sweet cherries (Table S1). *Aureobasidium pullulans*, *Taphrina wiesneri*, and *Metschnikowia crysoperlae* prevailed on both types of freshly harvested berries. The abundance of some yeast species was different on freshly collected sour and sweet cherries, e.g., *Hanseniaspora uvarum* was more abundant in PA-PL, while more *Cladosporium cladosporioides* sequences were detected in PC-PL than in PA-PL samples. Certain species were only found on one type of freshly harvested cherries, e.g., *Microcyclosporella mali*, *Ramularia citricola*, and *Neosetophoma rosae* were detected only in PC-PL samples (Table S1).

In store-purchased sour cherry samples, two phylum Ascomycota and Basidiomycota were distributed equally (the relative abundance was 51.37% and 48.43%, respectively) (Figure 3A). From the first phylum prevailed *Aureobasidium* (27.35%), *Hanseniaspora* (6.96%), and *Cladosporium* (6.46%) genera, and were mainly represented by *A. pullulans*, *H. uvarum*, and *C. cladosporioides* (Figure 3B, Table S1). The Basidiomycota were dominated by yeast from *Vishniacozyma* (23.32%), *Rhodotorula* (7.88%), and *Filobasidium* (6.10%) genera, with the most abundant species being *Vishniacozyma heimaeyensis*, *V. victoriae*, *Rhodotorula graminis*, and *Filobasidium wieringae* (syn. *Cryptococcus wieringae*) (Figure 3B, Table S1).

**Figure 4:**
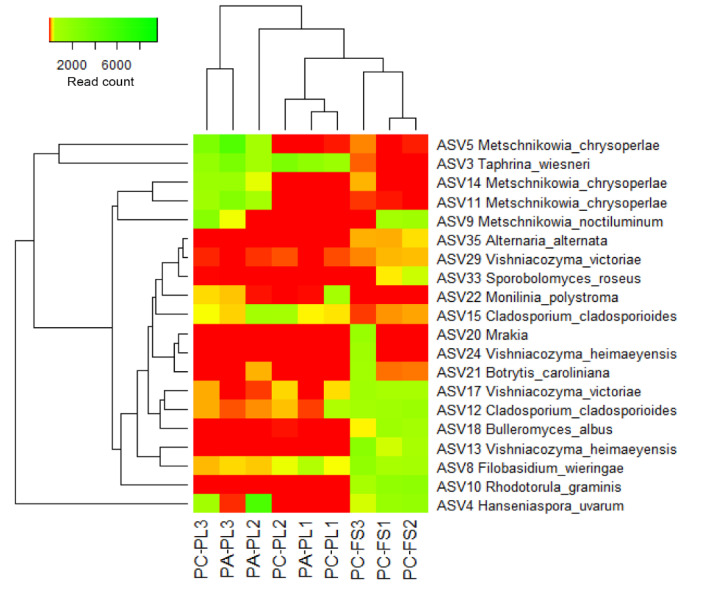
Heatmap of fungal unique amplicon sequence variants (ASVs) abundance on sour and sweet cherries. The color intensity is proportional to the relative abundance of fungi ASVs.

A heatmap illustrates the distribution of the most abundant fungal ASVs (Figure 4) revealing the differences between all three groups of berries—freshly harvested sour and sweet cherries, and sour cherries purchased from a local food store.

The core microbiomes detected in PC-PL, PA-PL, and PC-FS comprised of ASVs assigned to *A. pullulans* (Table S1). Based on the distribution pattern of dominating ASVs, PC-PL and PA-PL samples were separated from PC-FS samples, thus indicating on distinct community composition of freshly harvested and store berries. *M. crysoperlae* (ASVs 5, 11, and 14) and *T. wiesneri* (ASV 3) were highly associated with freshly harvested *P. cerasus* and *P. avium* berries. The abundance of ASVs representing *Rhodotorula* (ASV 10), *Filobasidium* (ASV 8), *Vishniacozyma* (ASVs 13 and 17), *Cladosporium* (ASV 12), and *Bulleromyces* (ASV 18) genera most likely conditioned clustering of a store sour cherries into a separate group. Some samples of sweet cherries demonstrated high similarity to sour cherries, for example, PC-PL3 and PA-PL3, PC-PL1 and PA-PL1 (Figure 4, Figure S2). This observation may indicate a similar ripening stage of freshly harvested *P. cerasus* and *P. avium* berries in particular samples.

### 3.3. Distribution of Cultivable Yeasts on P. avium and P. cerasus

A culture-based method was used to estimate viable yeast abundance and diversity on sweet and sour cherries. There was no noticeable difference in the overall quantity of yeasts between the samples of sour and sweet cherries collected from private plantations. The viable yeast population on the carposphere of freshly harvested sour cherries was 6.07 ± 0.31 log CFU/g and on sweet cherries was 5.93 ± 0.25 log CFU/g. However, sour cherries from a food store did exhibit about 10-fold higher CFU of yeasts per gram of berries (7.17 ± 0.12 log CFU/g) than freshly sampled berries. Representatives of phenotypically distinct colonies were randomly selected and applied for molecular identification by sequencing of the internal transcribed spacer ITS and/or D1-D2 domains of ribosomal DNA. According to the high similarity of generated sequences to those deposited in the GenBank database, yeast species were identified (Table S2).

**Figure 5:**
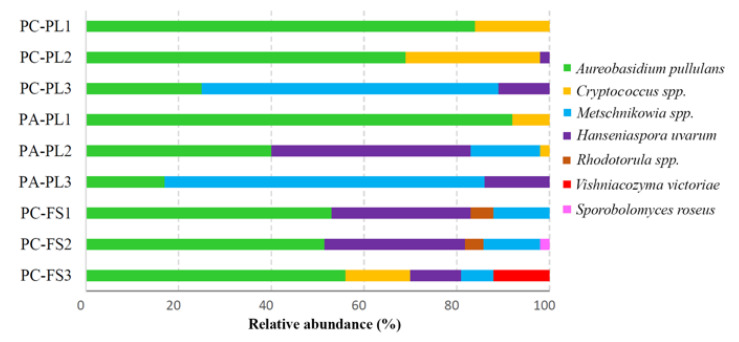
Relative abundance of yeasts isolated from the surface of sour and sweet cherries. Samples of: freshly harvested sour cherries—PC-PL, freshly harvested sweet cherries—PA-PL, food store sour cherries—PC-FS.

In total, 17 yeast species representing 10 genera were detected on freshly picked and food store samples (Table S2). Based on the diversity of isolated yeasts, sample-based variations in both types of berries were observed. *A. pullulans* yeasts were recovered from all tested samples and were the most dominant in PC-PL1 (84%), PC-PL2 (69%), and PA-PL1 (92%) samples (Figure 5, Table S2). Species from *Metschnikowia* genus (*M. pulcherrima, M. fructicola*, *M. sinensis*, and *M. viticola*) were detected on most tested berries with a high prevalence on freshly harvested PC-PL3 (64%) and PA-PL3 (69%) samples (Figure 5).

*Hanseniaspora uvarum* yeasts were isolated from all samples except for PC-PL1 and PA-PL1. The highest quantities of *H. uvarum* were detected on sweet cherries PA-PL2 (43%) and food store sour cherries in samples PC-FS1 and PC-FS2 (about 30% in both). The genus *Rhodotorula* was represented by the species *R. graminis*, *R. glutinis*, and *R. babjevae*, and formed 5–6% of the total yeast’s population on the food store cherries. Species from *Cryptococcus* genus (*C. wieringae* and *C. pinus*) were observed in sour cherry samples PC-PL2 (29%), PC-PL1 (16%), PC-FS3 (14%), and in sweet cherry PA-PL1 (8%). Some yeast species were not picked up by the direct dilution method and were only detected in low quantities after applying culture enrichment. These species include *Pichia kluyveri*, *Saccharomyces paradoxus*, *Saccharomyces cerevisiae*, and *Torulaspora delbrueckii* (Table S2).

### 3.4. Antagonistic Activity of Sour and Sweet Cherries-Associated Yeasts

Isolated yeasts were subjected to the analysis of killer activity. Out of 50 screened yeast strains (Table S2), 17 exhibited killing activity against *S. cerevisiae* strain BY4741 (Figure S3). The highest number of yeasts possessing killer features was isolated from PC-PL3 and PA-PL3 samples. They belong to *M. pulcherrima*, *M. fructicola*, *S. paradoxus*, *P. kluyveri*, and *H. uvarum* species. No killer yeasts were detected in samples of freshly harvested sweet and sour cherries (PC-PL1 and PA-PL1), and these samples showed low diversity of cultivable yeasts. Four killer yeast strains assigned to *M. fructicola*, *M. sinensis*, *T. delbrueckii*, and *H. uvarum* were recovered from the food store samples.

**Figure 6:**
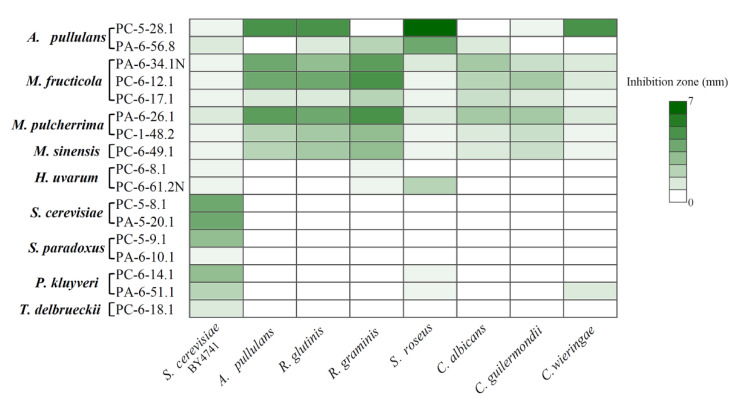
Antagonistic activity of yeasts inhabiting *P. cerasus* and *P. avium* surface.

The strongest killing ability against *S. cerevisiae* yeasts was demonstrated by *S. cerevisiae* PC-5-8.1 and PA-5-20.1, *S. paradoxus* PC-5-9.1, and *P. kluyveri* PC-6-14.1 strains (Figure 6). They formed 3-4 mm lysis zones. The moderate activity against *S. cerevisiae* yeasts was shown by *P. kluyveri* PA-6-51.1, *T. delbrueckii* PC-6-18.1, *A. pullulans* PA-6-56.8, and *M. pulcherrima* PA-6-26.1 yeasts. The weakest killing properties (inhibition zone size about 0.5 mm) showed *H. uvarum* PC-6-8.1 and PC-6-61.2N, *M. fructicola* PC-6-17.1, PA-6-34.1N, and PC-6-12.1, *M. pulcherrima* PC-1-48.2, *M. sinensis* PC-6-49.1, *S. paradoxus* PA-6-10.1, and *A. pullulans* PC-5-28.1 cultures (Figure 6).

Yeast strains with killing properties were used for the evaluation of antagonistic activity against fungal microorganisms from *Aureobasidium*, *Candida*, *Cryptococcus*, *Rhodotorula*, and *Sporobolomyces* genera (Figure 6, Figure S3). Some of these species may be attributed to potential pathogens [30,31,32,33,34,35]. The halo of inhibition was dependent not only on the species but also on the strain.

Among tested yeasts, *M. pulcherrima*, *M. fructicola*, and *M. sinensis* (in total six strains) exhibited the broadest spectrum of antagonistic activity by inhibiting the growth of all tested yeast species (Figure 6). The highest activity was demonstrated by *M. fructicola* PA-6-34.1N and PC-6-12.1, as well as *M. pulcherrima* PA-6-26.1 yeasts against *A. pullulans*, *R. graminis*, and *R. glutinis* species. Potential human pathogens *C. albicans* and *C. guilermondii* were also killed effectively by all tested *Metschnikowia* strains. The weakest antagonistic activity of *Metschnikowia* spp. strains were determined against *C. wieringae* and *S. roseus* species. *A. pullulans* strains PC-5-28.1 and PA-6-56.8 were active against six and five species, respectively. The highest killing activity of *A. pullulans* PC-5-28.1 strain was observed against *S. roseus*, *C. wieringae*, and non-killer *A. pullulans* strains. This yeast-like fungus was able to suppress the growth of potential human pathogens *R. glutinis* and *C. guilermondii.* The antagonistic activity of another *A. pullulans* strain PA-6-56.8 was detected against both *Rhodotorula* species, *C. albicans*, and *S. roseus* yeasts. *H. uvarum* PC-6-61.2N and *P. kluyveri* PA-6-51.1 strains demonstrated killing activity against three yeast species. In addition to antagonistic activity against *S. cerevisiae* and *S. roseus*, the *P. kluyveri* PA-6-51.1 strain also inhibits the growth of *C. wieringae*, while *H. uvarum* PC-6-61.2N acts against *R. graminis*. The rest of the tested yeast strains demonstrated antagonistic activity against one or two yeast species.

## 4. Discussion

To date, rather scattered information addresses microbial biodiversity on economically important *P. cerasus* and *P. avium* berries. Most of the studies are based on cultivation techniques and are focused on the trees- but not berry-associated microbiota [18,21,22]. In the present study, we characterize the composition of fungal communities deposited on sour and sweet cherries, using high-throughput DNA sequencing. To deepen insights into potentially advantageous or dangerous microorganisms inhabiting tested berries, NGS approach was supplemented with cultivation-based experiments. Our study provides valuable data on the differences in epiphytic mycobiota structure of environmental, freshly collected berries and those purchased in a food store.

On freshly harvested sour and sweet cherries, members of Ascomycota were found with higher frequency than those of Basidiomycota. Obtained results are in concord with the previous studies accomplished with fresh-cut fruits such as apple, red and black currants, sea buckhorn, etc. [14,16,17,23,36]. Ascomycetous fungi distributed on the tested berries were mainly represented by *Aureobasidium*, *Cladosporium*, *Metschnikowia*, *Hanseniaspora*, and *Taphrina* genera. It was previously demonstrated that intact berries were dominated by *Aureobasidium*, *Taphrina*, and *Cladosporium* fungi [14,37]. During the ripening process, fermentative ascomycetous microorganisms, such as *Metschnikowia*, *Candida*, and *Hanseniaspora* increased in frequency [14,37]. In our study, the heterogeneous microbial composition of the individual sweet and sour cherry samples was observed starting from the class level and this could be due to the uneven ripening stage of harvested berries. Increased distribution of *Metschnikowia* (in PC-PL3, PA-PL2, and PA-PL3 samples) and *Hanseniaspora* (in PA-PL2) genera yeasts suggests a higher ripening stage of berries in these samples, compared to other samples of freshly picked berries. At the same time, an increased level of *Aureobasidium* in PC-PL1 and PA-PL1 samples may be an indication of incomplete ripeness.

Among the most abundant genera inhabiting the surface of freshly picked sour and sweet cherries, fungi with previously reported beneficial biocontrol features were observed [13]. Epiphytic yeasts represent the greatest share of the fungal fruit-associated microbial community. They are successfully adapted to various environmental conditions and seem to be promising biological control candidates [38]. *A. pullulans* was the most abundant yeast-like fungus identified on the examined sour and sweet cherries. Numerous studies have revealed that some *A. pullulans* isolates due to the production of volatile organic compounds, enzymes, or toxins demonstrated antagonistic activity against various fungal and bacterial pathogens, such as *Botrytis*, *Bacillus*, *Colletotrichum, Geotrichum, Penicillium* [14,39,40,41]. In the present study, two strains of *A. pullulans* with a broad spectrum of antagonistic activity against potential phytopathogens or fungal microorganisms potentially harmful for humans were isolated. *Metschnikowia* spp. are sugar-rich fruit surface inhabiting species, found on apples, grapes, pears, raspberries, currants, strawberries, and plums [38,42,43]. These yeasts could be transported to new niches by insects and serve them as a food source [43,44]. In our study, *Metschnikowia* spp. yeasts were identified on the tested berries by NGS technique, and numerous species (such as *M. pulcherrima*, *M. fructicola*, *M. sinensis*, *M. viticola*) were isolated using plate dilution assay. It is known that some strains of *M. pulcherrima*, *M. fructicola*, and *M. sinensis* have a strong biocontrol activity against various microorganisms, due to the synthesis of volatile organic compounds, iron immobilizing pigment pulcherrimin, or killer toxins [38,45,46,47,48,49,50]. These yeasts are of the greatest interest as potential inhibitors of pathogenic microorganisms [38]. About half of *Metschnikowia* spp. strains isolated in our study also exhibited pronounced antagonistic activity against all tested fungal microorganisms, thus could be attractive for disease management. *Hanseniaspora* spp. have been frequently found on mature fruits such as apple, grape, plum, and sea buckthorn [14,43,51]. Some *Hanseniaspora* genus species, especially *H. uvarum*, display industrially significant antagonistic properties against fungi pathogenic to humans (*Trichophyton mentagrophytes* and *T. rubrum*) and plants (*Botrytis cinerea*, *Colletotrichum capsica*, *Fusarium solani*, *Penicillium* spp. and *Sclerotinia sclerotiorum*) [52,53,54]. Antimicrobial activity against pathogenic bacteria *Staphylococcus aureus*, *Escherichia coli*, *Klebsiella pneumonia*, and *Pseudomonas aeruginosa* was attributed to *H. uvarum* encoded killer toxin [54]. In our study, two isolated *H. uvarum* strains exhibited killing activity against *R. graminis* and *S. cerevisiae* yeasts, demonstrating their biocontrol potential. The members of Saccharomycetes, such as *P. kluyveri*, *S. cerevisiae*, *S. paradoxus*, and *T. delbrueckii*, were detected on sweet and sour cherries at a low level. Our observation agrees with previous studies performed on apple, plum, grape, and pear fruits [43,55,56]. Even at low quantities such yeasts could act as biocontrol agents and regulate the structure of plant microbiota [17].

The number of fungi from Basidiomycota phylum was higher in food store sour cherries and reached a similar level as Ascomycota representatives, and may be related to both fruit and postharvest handling-dependent occurrence. It is well established that various treatments and conditions can affect the composition of the microbial community [57]. Postharvest processing of fruits, packaging, and distribution may condition microbial contamination harmful for consumer health [18]. The abundance of microorganisms can be decreased or avoided by good harvesting and handling practice, cool storage of fruits, and usage of chemicals or fungicides [18].

We observed a high frequency of potentially pathogenic fungi from the genera of *Rhodotorula*, *Vishniacozyma*, *Filobasidium* (syn. *Cryptococcus*), and *Sporobolomyces* on the purchased sour cherries. We demonstrated that isolated strains from the mentioned above species did not possess any antimicrobial activity against tested microorganisms. *Rhodotorula* spp. are ubiquitous yeasts that can be recovered from many environmental sources, including fruits. Some *Rhodotorula* species, such as *R. graminis*, *R. mucilaginosa*, and *R. babjevae* award growth benefits to plants, enrich their biochemical properties [58,59,60]. *Rhodotorula* yeasts exhibit strong biocontrol activity against fungal and bacterial pathogens, especially towards *B. cinerea* and *P. expansum* [59]. On the other hand, some species such as *R. mucilaginosa*, *R. glutinis*, and *R. minuta* have emerged as opportunistic pathogens that can infect humans with the weakened immune system and cause a variety of systemic infections [35]. Some *Sporobolomyces* and *Vishniacozyma* species showed biocontrol advantages by reducing the growth of blue and gray mold [61,62]. On the other hand, *S. roseus* yeasts are documented as one of the yeasts involved in the candied fruit and nougat spoilage, thus rendering inconvenience for the food industry [30]. The danger is even more sound considering that these species may cause infection in immunocompromised patients [32]. *Cryptococcus* spp. are typical members of the yeast community on fruits at an early stage of maturation [43,63]. These ubiquitous fungi enclose species producing biocontrol agents against many pathogens [13,64], while particular species can be dangerous for humans [31].

Our findings suggest that fungal communities on the surface of sour cherries purchased from a food store inhabit more controversial yeast genera than the freshly picked berries in terms of described yeasts that have attractive or undesirable properties for food safety and human health. We have also observed stronger antagonistic properties against tested yeast species of strains isolated from freshly picked cherries than in the samples of store berries. The examination of fungal communities of *P. cerasus* L. and *P. avium* L. fruits enabled the isolation of various yeast strains with a high biocontrol potential and further research is required to better reveal the value of these microscopic fungi.

## 5. Conclusions

This is the first manuscript characterizing fungal communities on sweet and sour cherry fruits by applying NGS approach and cultivation-based techniques. Our data show the plant-conditioned prevalence of microorganisms and demonstrate the influence of postharvest handling, such as food store management, on the structure of mycobiota. Investigation of microbial ASVs diversity showed a clear separation of fungal assemblages on freshly harvested and food store cherries. Among the fungi inhabiting tested berries, potentially beneficial or pathogenic fungi were documented. Numerous cultivable yeasts were isolated from the surface of tested berries and evaluated for their antagonistic activity. The prominent antagonistic activity of cultivable yeasts isolated from freshly harvested cherries against potential pathogens highlights the attractiveness of these strains for the management of disease control.

## Figures and Tables

**Table 1:** Total sequences obtained for fungal community samples of sour (PC-PL) and sweet (PA-PL) cherries freshly harvested from private plantations as well as food store sour cherries (PC-FS).

	Reads Obtained	HQ Reads	Observed ASVs	Pielou Evenness	Shannon Diversity	Simpson Index
PC-PL1	26,895	17,126	99	0.63	4.2	0.86
PC-PL2	28,665	19,082	109	0.66	4.46	0.91
PC-PL3	30,732	21,140	109	0.65	4.37	0.92
PA-PL1	27,571	20,325	71	0.45	2.75	0.72
PA-PL2	28,473	17,509	52	0.54	3.08	0.78
PA-PL3	29,021	19,901	71	0.58	3.59	0.86
PC-FS1	27,825	17,104	46	0.65	3.58	0.85
PC-FS2	30,369	19,242	58	0.65	3.78	0.87
PC-FS3	28,433	17,504	75	0.68	4.26	0.92
Total:	257,984	168,933	690			

## Data Availability

Not applicable.

## Supplementary Materials

The following are available online at https://www.mdpi.com/article/10.3390/microorganisms9071423/s1, Figure S1: Rarefaction curves at a genetic distance of 3% for each sample; Figure S2: Fungal taxonomy relative abundance at phylum (A) and genus (B) level for samples of freshly harvested sweet and sour cherries as well as sour cherries from the food store; Figure S3: Yeast antagonistic activity assay; Table S1: Fungal communities associated with freshly harvested and food store-purchased *Prunus* *cerasus* and *Prunus avium* berries; Table S2: Yeast isolates detected in this study.
