# Supplementary figures and images for: Mycobiota in the Carposphere of Sour and Sweet Cherries and Antagonistic Features of Potential Biocontrol Yeasts
Source: Microorganisms. 2021 Jun 30;9(7):1423. doi: 10.3390/microorganisms9071423 (PMC8307871; doi:10.3390/microorganisms9071423)

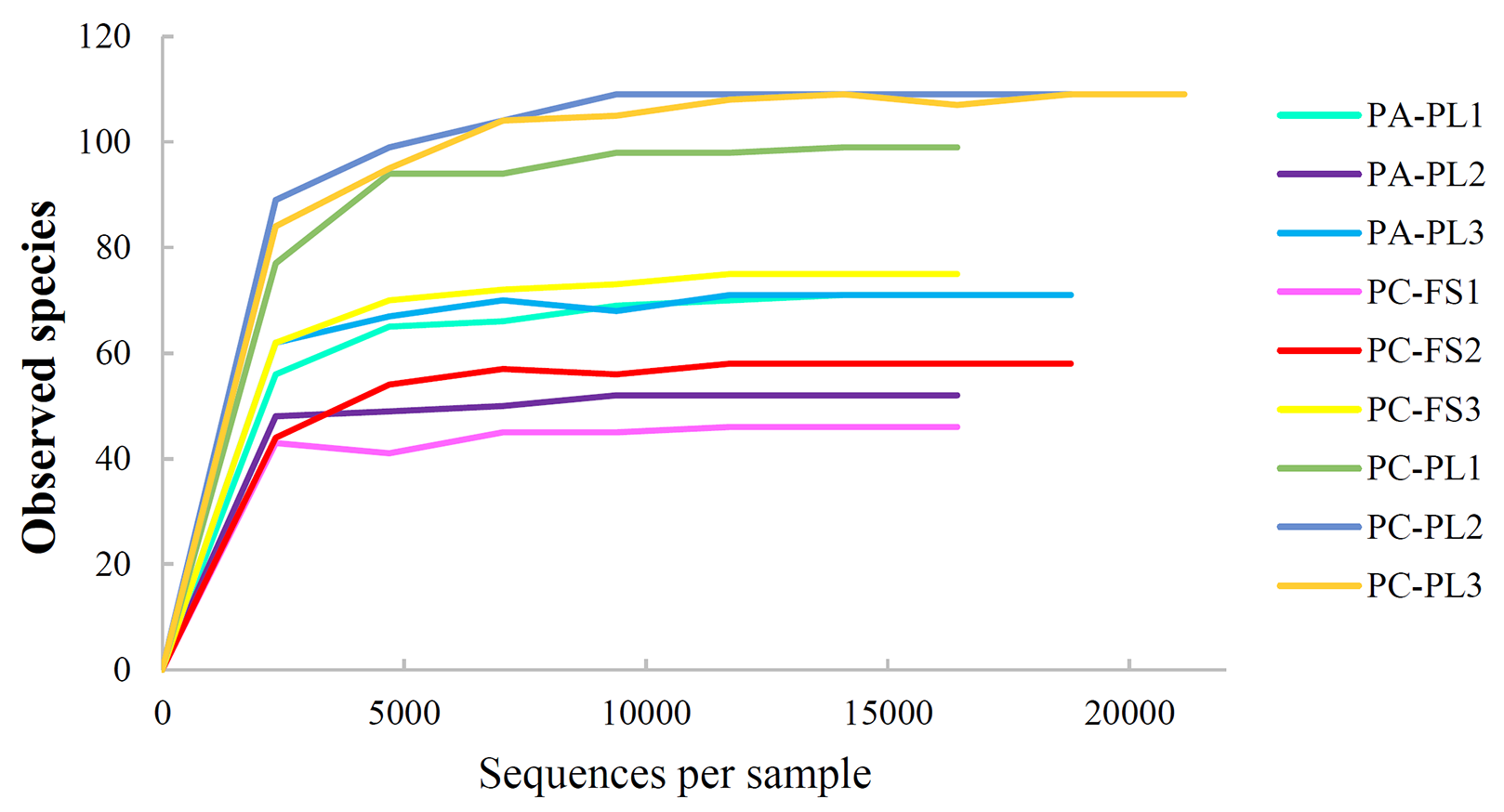

Supplement: Supplementary file 1 [file microorganisms-09-01423-s001.zip › Figure S1.tif]

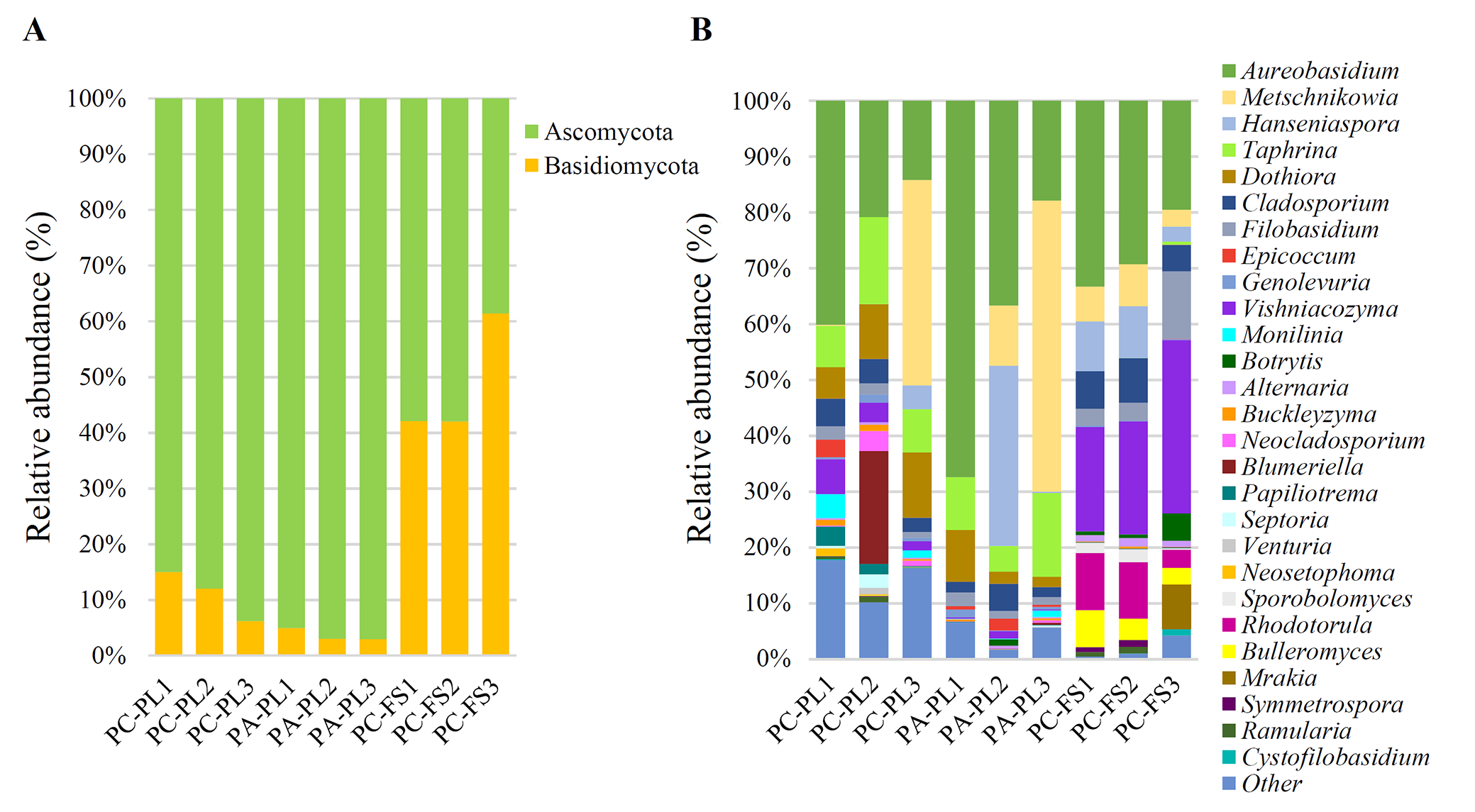

Supplement: Supplementary file 1 [file microorganisms-09-01423-s001.zip › Figure S2.tif]

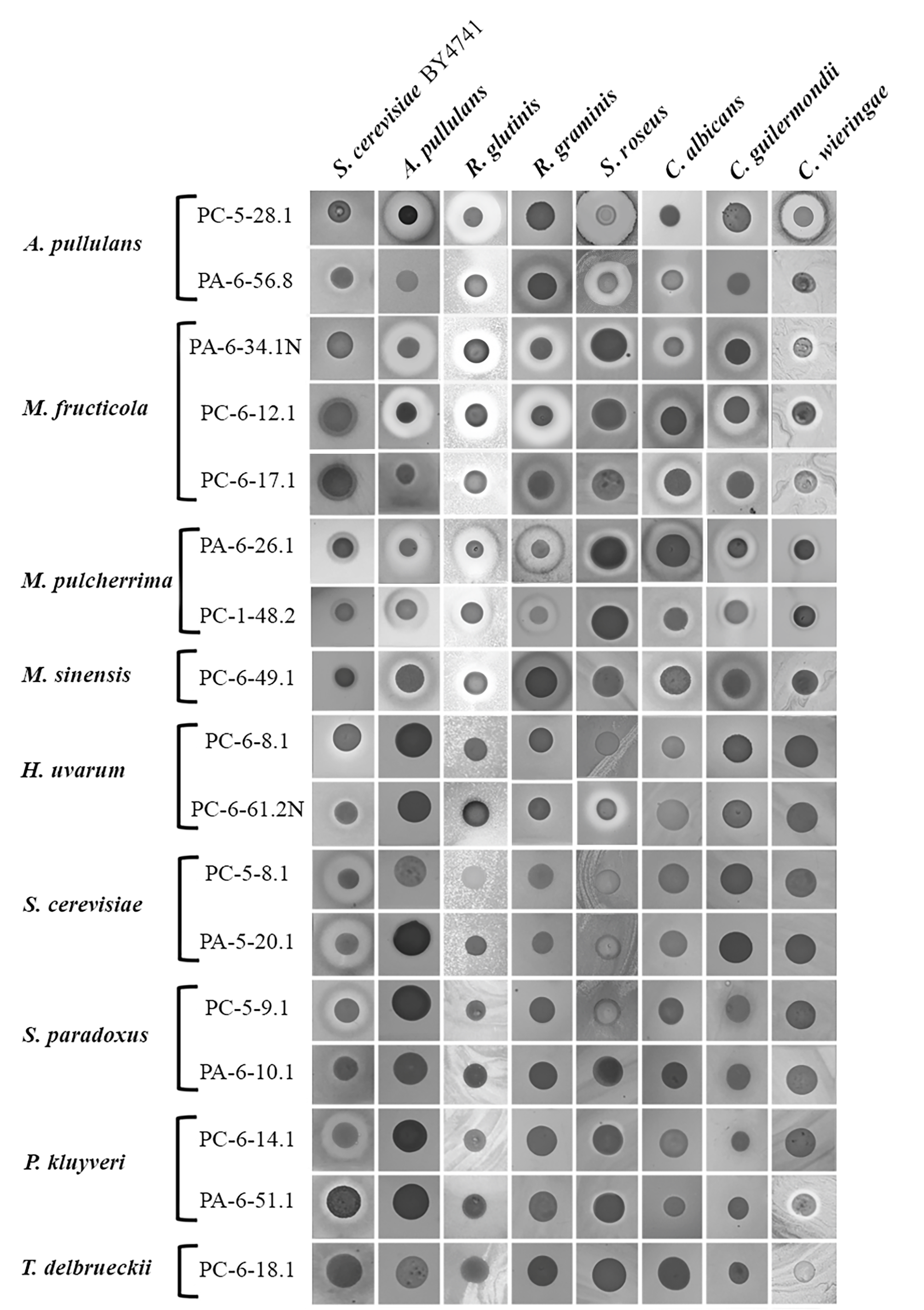

Supplement: Supplementary file 1 [file microorganisms-09-01423-s001.zip › Figure S3.tif]
